# Diabetic Retinopathy Detection Using Local Extrema Quantized Haralick Features with Long Short-Term Memory Network

**DOI:** 10.1155/2021/6618666

**Published:** 2021-04-14

**Authors:** Abubakar M. Ashir, Salisu Ibrahim, Mohammed Abdulghani, Abdullahi Abdu Ibrahim, Mohammed S. Anwar

**Affiliations:** ^1^Department of Computer Engineering, Tishk International University, Erbil, KRD, Iraq; ^2^Department of Mathematic Education, Tishk International University, Erbil, KRD, Iraq; ^3^Department of Computer Engineering, Altinbas University, Istanbul, Turkey

## Abstract

Diabetic retinopathy is one of the leading diseases affecting eyes. Lack of early detection and treatment can lead to total blindness of the diseased eyes. Recently, numerous researchers have attempted producing automatic diabetic retinopathy detection techniques to supplement diagnosis and early treatment of diabetic retinopathy symptoms. In this manuscript, a new approach has been proposed. The proposed approach utilizes the feature extracted from the fundus image using a local extrema information with quantized Haralick features. The quantized features encode not only the textural Haralick features but also exploit the multiresolution information of numerous symptoms in diabetic retinopathy. Long Short-Term Memory network together with local extrema pattern provides a probabilistic approach to analyze each segment of the image with higher precision which helps to suppress false positive occurrences. The proposed approach analyzes the retina vasculature and hard-exudate symptoms of diabetic retinopathy on two different public datasets. The experimental results evaluated using performance matrices such as specificity, accuracy, and sensitivity reveal promising indices. Similarly, comparison with the related state-of-the-art researches highlights the validity of the proposed method. The proposed approach performs better than most of the researches used for comparison.

## 1. Introduction

Diabetic retinopathy (DR) is a medical term used to describe a sightly threatening ailment which appears on the retina. The disease is predominantly common among the working age people which when properly not treated could lead to total loss of vision [1, 2]. Retinopathy is the leading cause of blindness amongst adults worldwide [3]. According to a research, over 239 million people in the year 2010 were affected with over 7.7 million in America alone. The number of Americans with diabetic retinopathy, according to the new projection, is expected to nearly double, from 7.7 million in 2010 to around 14.6 million by the year 2050 [4]. Sequel to its enormity and the gravity of devastation it causes, early detection and treatment has been one of the key focus for most health institutions around the world.

Diabetic retinopathy causes major changes in retina vasculature structure which comprises the major blood vessels used for transforming oxygenated blood and nutrients to the various part of the retina. In some people with diabetic retinopathy, blood vessels may swell up and leak fluid leading to formation of abnormalities such as hard and soft exudates. In other situations, development of abnormal new blood vessels, blood vessels occlusion, and leakage of blood (hemorrhages) into a healthy portion of the retina are experienced. Detection of the signs of diabetic retinopathy involves proper identification of all the possible abnormalities such as hard and soft exudates, hemorrhages, and blood vessel occlusions [2, 3, 5].

Therefore, an effective technique to realize a reasonable diagnosis of DR diseased eye will involve identification and segmentation of the various features of the retina such as blood vessels, optic disk, and many other anomalous symptoms of a diseased eye. Segmentation of blood vessels is one of the important aspects being looked at, whereby the retina vasculature is being extracted from the fundus image for close examination of some anatomical changes in the structures of these vessels such as diameter, branching angle, and occlusion.

**Figure 1:**
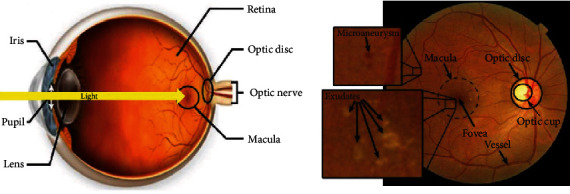
Anatomical view of retina.

In the past, diagnosis of these retinal symptoms depends on a manual segmentation of the retinal fundus image. This action requires an ophthalmologist expertise as the procedure is time-consuming, effort prone, and very complicated [6]. However, with the major breakthrough in machine learning and artificial intelligence fields, many algorithms were developed by researchers to help accomplish the process of diagnosis of these abnormalities in retina fundus images. For example, ophthalmologists can use a properly segmented vessel to examine and detect a disease by identifying the growth of number of extra vessels or fluids like features, their shapes, and sizes. Different techniques have been adopted or proposed in many researches, some of which focused mainly on the segmentation of various features of the retina to examine anomalies. Moreover, some techniques have more general approach, whereas machine learning algorithm is developed and trained to classify a fundus image as healthy or nonhealthy with various stages of the disease. Anatomical overview of retina is depicted in Figure 1.

The manuscript is divided into seven sections. Sections 1 and 2 comprise of the introduction and literature review while in Section 3, theoretical background of the key concepts used in the proposed approach was presented. In Section 4, the proposed approach was discussed while Section 5 presents the experimental results using the proposed approach. In Section 6, discussion and comparison were presented, and the manuscript was concluded in Section 7.

## 2. Related Literature

Quite recently, a number of researches have emerged giving birth to computer-aided diagnostic systems which are used for diagnosis of ophthalmic anomalies in the retina fundus images. Blood vessel segmentation has seen one of the highest volumes of articles produced [2, 7]. Most of the techniques used in the segmentations can be grouped into two categories: supervised methods and unsupervised method. The supervised methods use labeled data to train classifier algorithms such as support vector machine (SVM) to classify each pixel according to the labels. The unsupervised methods in contrast use no label data or any prior information about the disease. The unsupervised techniques may include morphological operation, matched filtering approach, and deformable models [1].

Yin et al., [8] used probabilistic tracking-based method to segment the retina blood vessel from the fundus images using structural Analysis for Retina (STARE) and Digital retinal Images for a Vessel Extraction (DRIVE) databases. Sensitivity and specificity of 0.75 and 0.95 were recorded, respectively.

In their submission, Ravichandran and Raja [9] applied local entropy-based thresholding technique after preprocessing the fundus image. Wang et al., [10] utilized supervised learning approach in which the Convolutional Neural Network (CNN) and Random Forest (RF) were combined to classify vessel pixel after being trained with a label data. This method performed well but at a cost of huge computational cost incurred.

Sohini et al., [11] extracted major vessels in the preprocessing stage, and then, they applied Gaussian Mixture model (GMM) classifier has been used tune and refine the final vessel from the rest of the image. Zhao et al. [12] used active contour techniques by applying graph cut to segment vessels. They initially enhanced the image using local phase filter. Zhao et al. [13] proposed a new infinite active contour model for the automated detection of retinal blood vessels.

Walter et al. [14] introduced an algorithm for microaneurysm detection using candidate extraction. Their techniques initially enhance the image and then extract the green channel and normalize it followed by candidate detection with diameter closing and an automatic thresholding scheme. The classification of the microaneurysm pixel candidates was done based on kernel density estimation.

Similarly, Spencer et al. [15] and Frame et al. [16] both applied candidate extractor approach where shade correction was used by subtracting a median filtered background from the green channel image. Then, they finally used morphological operation based on top-hat transformations using twelve structuring elements to extract candidate extraction. The resulted candidate pixels were further subjected to contrast enhancement operations before finally were binarized.

## 3. Theoretical Background

### 3.1. Gray-Level Cooccurrence Matrix

**Figure 2:**
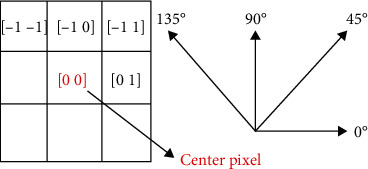
GLCM center pixel neighborhoods offset.

GLCM is commonly used in texture analysis and was first introduced by Haralick et al. in 1973 for extracting statistical textural features from an image [17]. Haralick texture features calculated from GLCM encode important texture descriptors which can be used for texture classification and region of interest (ROI) localization in an image. The GLCM is constructed by considering each pixel in relation to its neighborhood pixels. It gives the distribution of cooccurring pixel values for a particular neighborhood under consideration. The neighborhood of the center pixel is defined in terms of their distance and direction (offset) from the center pixels [18]. An offset of [−1, −1] or [1,135°] describes a neighborhood pixel at one-pixel distance (*d* = 1) and 135 degrees (*θ* = 135°) from the center pixel.  Figure 2 shows how these offsets are considered.

Therefore, for a gray image *I* of size *M* × *N* with *L* gray-level values, the cooccurrence matrix *C* will have a size *L* × *L* which is define over the entire image and parameterized by the offset (*d*, *θ*).

(1) $$\begin{array}{c} C_{d,\theta }\left(i,j\right)=\overset{M}{\underset{m=1}{\sum }}\overset{N}{\underset{n=1}{\sum }}\left\{\begin{array}{c} 1,\;\text{if}I\left(m,n\right)=i\text{ and }I_{d,\theta }\left(m,n\right)=j,\\ 0,\text{ otherwise}. \end{array}\right. \end{array}$$

GLCM computed at the four different offsets defined by offset angle [0°,45°,90°,135°] typically encodes transition value information on both the horizontal, vertical, and two diagonals. Such GLCM is rotation invariant. Symmetrical GLCM is formed when all offsets on the opposite symmetry of the four directions are considered in constructing the GLCM (i.e., all directions are considered) [18].

**Figure 3:**
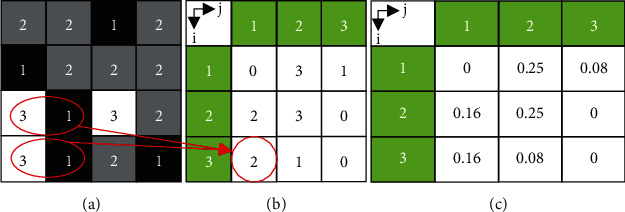
GLCM computation. (a) Original gray image with 3 gray levels, (b) computed GLCM using (*d* = 1, *θ* = 0°) offset, and (c) normalized GLCM which sums up to one.

Consider Figure 3(a) of an original gray image with gray level *L* = 3, if a horizontal offset is defined (i.e., *d* = 1, *θ* = 0°), the computed GLCM in Figure 3(b) is a 3 × 3 sized whose entries are computed using Eq. (1). For instance, the highlighted entry “2” of the GLCM matrix (Figure 3(b)) which occurs at row  (*i* = 3) and column (*j* = 1) was obtained by counting the number of pixel pair on the horizontal offset which have gray values exactly 3 and 1, respectively, from the original gray image in Figure 3(a). The normalized GLCM in Figure 3(c) represents the estimated probability of each combination of GLCM occurrence to occur within the image. For each row of the gray image in Figure 3(a), there are 3 unique possibilities for combination of pixel pair on the horizontal offset; therefore, a total of 12 possibilities exist for the entire gray image. The normalized GLCM in Figure 3(c) is obtained by dividing each gray cooccurrence entries in Figure 3(b) by 12. This normalized GLCM is used to extract Haralick features, and its entries sum up to one. The normalized GLCM can be considered as a probability mass function of the gray-level pairs in the image.

### 3.2. Haralick Textural Features

Haralick computed 24 different statistical features from the normalized GLCM matrix, *C*_*n*_ as exemplified in Figure 3(c). These features quantify essential part of local information and spatial features within the image. Though all the 24 features may be useful in different textural analyses, in this research after a few trial combinations of these features to extract the desired information, five of these features are standout to very promising hence are described in Eqs. (2)–(6).

*Homogeneity*: describes the measure of closeness of the each GLCM element to its diagonal elements

(2) $$\begin{array}{c} \text{Homogenity}=\overset{M}{\underset{m=1}{\sum }}\overset{N}{\underset{n=1}{\sum }}\frac{C_{n}\left(i,j\right)}{1+{\left(i-j\right)}^{2}}. \end{array}$$

*Entropy*: is the measure of randomness or the degree of disorder present in the image

(3) $$\begin{array}{c} \text{Entropy}=-\overset{M}{\underset{m=1}{\sum }}\overset{N}{\underset{n=1}{\sum }}C_{n}\left(i,j\right)\ln C_{n}\left(i,j\right). \end{array}$$

*Energy*: is the root of Angular Second Moment, which gives the measures of the local uniformity of the gray levels

(4) $$\begin{array}{c} \text{Energy}=\sqrt{\overset{M}{\underset{m=1}{\sum }}\overset{N}{\underset{n=1}{\sum }}{C_{n}}^{2}\left(i,j\right)}. \end{array}$$

*Correlation*: this feature shows the linear dependency of gray-level values in the cooccurrence matrix.

(5) $$\begin{array}{c} \text{Correlation}=\overset{M}{\underset{m=1}{\sum }}\overset{N}{\underset{n=1}{\sum }}C_{n}\left(i,j\right)\frac{\left(i-\mu_{x}\right)\left(j-\mu_{y}\right)}{\sigma_{x}\sigma_{y}}, \end{array}$$

where terms *μ*_*x*_*μ*_*y*_ and *σ*_*x*_ *σ*_*y*_ are the means and standard deviation of the summed cooccurrence matrix *C*_*n*_, a long horizontal and vertical spatial plane, respectively.

*Contrast*: contrast which is also known as standard deviation indicates the measure of gray-level intensity variation between pixels.

(6) $$\begin{array}{c} C=\overset{M}{\underset{m=1}{\sum }}\overset{N}{\underset{n=1}{\sum }}{\left(i-j\right)}^{2}C_{n}\left(i,j\right). \end{array}$$

### 3.3. Long Short-Term Memory (LSTM) Network

LSTM network is a deep learning form of Recurrent Neural Network (RNN) which was first proposed in 1997 by Hochreiter and Schmidhuber [19]. It addresses the familiar problems of vanishing/exploding gradients associated with traditional neural networks. The vanishing gradient problem gradually erodes the magnitude of error gradients used to update weights and biases. This prevents the network from further adjusting weights and biases, and hence, the learning eventually halts in the deeper layers of the network [13].

**Figure 4:**
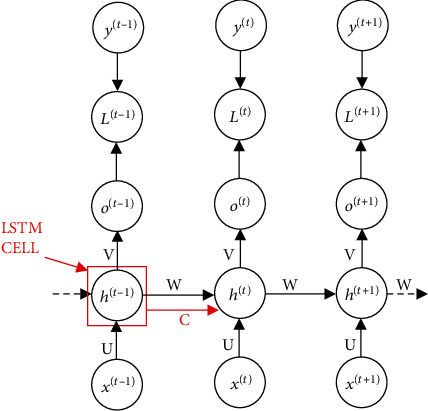
Recurrent Neural Network.

In a classical structure of RNN (Figure 4), the network updates the weight vectors **W**, at each hidden layer. The out of the hidden layer *h*_*t*_  at time stamp *t* is compared with data label *y*^(*t*)^ to compute the net error *L*^(*t*)^ for that layer which is used by gradient decent to minimize the network error. The net input to each hidden layer at time lag *t* comprises of the input sequence *x*^(*t*)^ and the weighted output (Wh^(*t* − 1)^) of the adjacent hidden layer at time *t* − 1.

**Figure 5:**
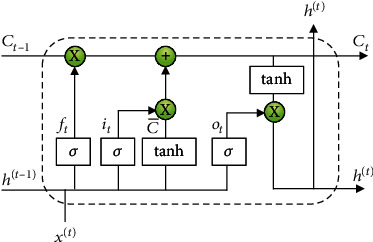
Long Short-Term Memory cell.

LSTM replaces each hidden layer by a gated structure called *cell* (Figure 5) which has additional connection to each layer using cell state, *C*. The LSTM cell consists of three gates: *forgetting gate*, *input gate*, and the *output gate*. Each of these gates uses sigmoid (*σ*) activation to control the amount of information through the cell at various stages. Forgetting gate *f*_*t*_, controls the information to retain or forget from the previous time lag of the sequence in the adjacent layers while the input gate *i*_*t*_ regulates the current internal cell state $\bar{C}$ which holds the cell's net input after being squashed using tanh activation function. The output gate *o*_*t*_, controls the cell output *h*^(*t*)^, which literally is a squashed vector of the current cell state *C*_*t*_ regulated by the output gate. The output gate controls the squashed current cell state.

The relations for LSTM network for each gate with biases can be presented in Eq. (7).

(7) $$\begin{array}{c} \begin{array}{c} f^{\left(t\right)}=\sigma \left(\left[w^{f}h^{\left(t-1\right)}+x^{\left(t\right)}\right]+b^{f}\right)\\ i^{\left(t\right)}=\sigma \left(\left[w^{i}h^{\left(t-1\right)}+x^{\left(t\right)}\right]+b^{i}\right)\\ o^{\left(t\right)}=\sigma \left(\left[w^{o}h^{\left(t-1\right)}+x^{\left(t\right)}\right]+b^{o}\right)\\ {\bar{C}}^{\left(t\right)}=\tanh\left[w^{c}h^{\left(t-1\right)}+x^{\left(t\right)}\right]\\ C^{\left(t\right)}=C^{\left(t-1\right)}f^{\left(t\right)}+i^{\left(t\right)}{\bar{C}}^{\left(t\right)}\\ h^{\left(t\right)}=\tanh\left[C^{\left(t\right)}\right]o^{\left(t\right)} \end{array} \end{array}$$

The LSTM network uses the gradient decent with truncated Backpropagation Through Time (BPTT) to adjust the weights (*w*^*f*^, *w*^*f*^, *w*^*f*^, *w*^*f*^)  and biases (*b*^*f*^, *b*^*i*^, *b*^0^) to minimize the error between the network's outputs and target outputs.

## 4. Proposed Method

In the proposed method, feature encoding uses the local extrema information at different quantization levels to construct the GLCM. The constructed normalized GLCM is used to extract Haralick features of interest which are subsequently encoded as a feature sequence to be fed to the LSTM network for training.

### 4.1. Local Extrema Quantized Haralick Features

It obvious that, for the same image, Haralick feature magnitudes are influenced by the number of discernible gray levels in the image, since number of gray levels determine the size of the GLCM. One way to seeing the effect is through quantization of the original gray level to new level. For a gray-level image, *I*, the quantized value is nonlinear transformation which presents the image at a different resolution than the original. Apart from the gray quantization level, other features like GLCM *offset* and predetermined gray-level intensity range (extrema) of the original gray image all affect Haralick features. These attributes are usually referred to as GLCM construction parameters

To capture two of these GLCM construction parameters, quantization level and extrema range of gray intensity image, GLCM is computed with a new image created from the original image *I*, with different quantization level (resolution) and local extrema range.

An image *I*, which is quantized at gray level *L*_*Q*_ with gray local extrema information *G*_*r*_ = [*I*_max_, *I*_min_] where *I*_max_, *I*_min_ represent the minimum and maximum of the local extrema gray-level intensity values, will have different Haralick features when these parameters are varied. The new modified image *I*_*s*_ which captures both quantization *L*_*Q*_  and *G*_*r*_ can be computed using Eqs. (8) and (9).

(8) $$\begin{array}{c} I_{\mathrm{int}}\left(m,n\right)=\left\lceil \frac{I\left(m,n\right)-I_{\min}}{I_{\max}-I_{\min}}\times L_{Q}\right\rceil , \end{array}$$

(9) $$\begin{array}{c} I_{s}\left(m,n\right)=\left\{\begin{array}{c} 1\;\text{if}I_{\mathrm{int}}\left(m,n\right)\leq 1,\\ L_{Q}\;\text{if }I_{\mathrm{int}}\left(m,n\right)\geq L_{Q},\\ I_{\mathrm{int}}\left(m,n\right)\text{ otherwise}, \end{array}\right. \end{array}$$

where *I*_int_ is an intermediary image and ⌈⌉ is a ceiling operator that maps the computed values in *I*_int_ to the least integer greater than or equals itself.

**Figure 6:**
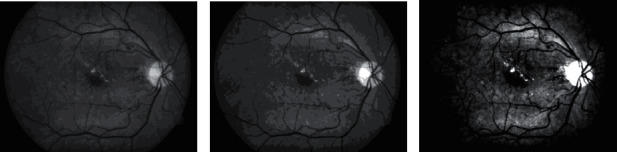
(a) Green channel of a retina fundus image after being preprocessed with adaptive histogram equalization. (b) Quantized version of (a) using *L*_*Q*_ = 8 and global extrema *G*_*r*_ = [2, 222]. (c) The same quantization level with (b) but with *G*_*r*_ = [48, 143] localized to a ROI containing hard exudate.

One of the common choices for the extrema *G*_*r*_ is the global minimum and maximum of the gray-intensity values in the image. A choice of *G*_*r*_ within a localized ROI in the image at the same quantization level *L*_*Q*_ will result in different intensity value distributions within the image compared to the same image whose *G*_*r*_ is the global extrema intensity values of the image. Hence, choice of extrema value *G*_*r*_ influences the GLCM matrix (see Figure 6).

The proposed quantized Haralick features are formed using the five Haralick features of the normalized GLCM as described in Section 3. Initially, four different versions of the original gray image are computed at different quantization levels, i.e., *L*_*Q*_ = [128, 64, 16, 8] bins.

(10) $$\begin{array}{c} I=\left\{I_{128},I_{64}\;I_{16}\;I_{8}\right\}. \end{array}$$

From each of the quantized version *I*_*Q*_, a normalized GLCM is constructed and then use to extract the five Haralick features (i.e., *homogeneity*, *entropy*, *energy*, *correlation*, and *contrast*). For each quantized image, minimum and maximum pixel intensity values are used as the extrema *G*_*r*_. For instance, the extrema values for *I*_128_ is given by (11).

(11) $$\begin{array}{c} G_{r_{127}}=\left[\min\left(I_{128}\right),\max\left(I_{128}\right)\right]. \end{array}$$

In the end, all the Haralick features extracted from the normalized GLCM of the four quantized images are concatenated to form a feature vector *F*_Haralic_  of length 20.

### 4.2. Segmentation

**Figure 7:**
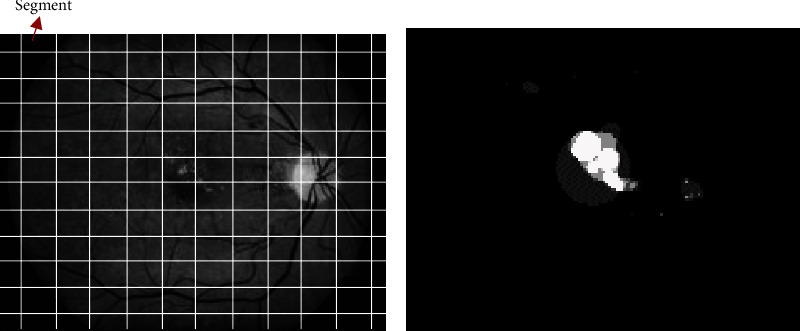
(a) Segmented training image using 64 × 64 window and (b) ground-truth of (a) with candidate ROI annotated for hard-exudate symptom.

Instead of applying the proposed feature selection described above to the entire gray fundus image, the image is segmented into 64 × 64 window. Where necessary, the image is padded with zeros to ensure that an integer number of segments of size 64 × 64 is generated. Each segmented window uses the proposed approach to form the 20 features. The objective of segmentation is to help find the probability of occurrence of region of interest (ROI) where the diabetic retinopathy symptoms most likely occur. To compute this probability, a prior information about the location of the symptoms is needed. This information is provided in the training datasets where regions that are infected were marked by experts. These probabilities for each segment are determined using a benchmarking method similarity measures between the features of a segment and the features of an actual ROI are compared. The ROI is simply the ground-truth image of the training data as indicated in Figure 7(b).

### 4.3. Sequence Encoding for LSTM

LSTM network is trained in a vector to sequence model. For any training image, its corresponding ground-truth is used to extract the 20 features proposed. If there are *N* training samples, each ground-truth (ROI) generates *F*_*i*_ *f*or *i* = 1 to *N* of length 20. The benchmark ROI feature vector *F*_ROI_  use to evaluate each segment is given as the average of these features as in (12).

(12) $$\begin{array}{c} F_{\text{ROI}}=\frac{1}{N}\overset{N}{\underset{i=1}{\sum }}F_{i}. \end{array}$$

A correlation coefficient *ρ* is computed between each *F*_ROI_ features and *F*_segment_  features to assign a label to that segment. To generate level for each segment with encoded feature sequence, a correlation coefficient *ρ* is computed between each encoded segment features and the encoded feature for the candidate ROI. If the computed *ρ* is less than 0.5, that segment is assigned a label of *none*. A value of *ρ* between 0.5 and 0.7 is labeled *mild*, 0.7 to 0.89 are labeled *strong*, and 0.9 to 1 take *very strong* label.

**Figure 8:**
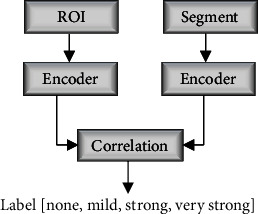
Label generation for LSTM.

Every segment extracted features (20 features) in the image is train as sequential input to the LSTM and the computed label as the label data. Therefore, the network trains on a stream of sequential data. For a ROI encoded feature *X* and segment feature *Y*, the correlation coefficient *ρ* is computed using Eq. (13). The complete flowchart of the proposed method is depicted in Figure 8.

(13) $$\begin{array}{c} \rho_{X,Y}=\frac{E\left[\left(X-\mu_{x}\right)\left(Y-\mu_{y}\right)\right]}{\sigma_{xi}\sigma_{y}}, \end{array}$$

where *E*[], *μ*, and *σ* are the expected value mean and variance functions, respectively.

**Figure 9:**
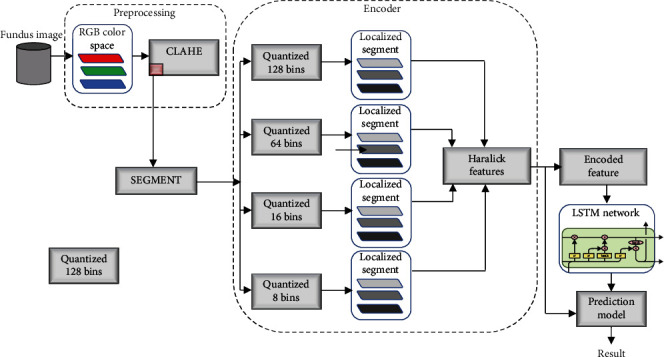
Flowchart of the proposed approach.

The LSTM network is trained with segments within each training data and their corresponding labels computed (correlation coefficient *ρ*) above. During testing, the network uses its learned model to predict the correlation coefficient *ρ* of a new segment presented to it. The result of the prediction which is interpreted as the probability of occurrence of the symptoms within that segment is used in subsequent stages to analyze and detect portion of the fundus image with symptoms. The complete flowchart of the proposed method is shown in Figure 9.

## 5. Experimental Results

The proposed approach was implemented on two separate popular public retinopathy datasets: *STARE* [20] and *Image Ret* datasets [21]. The *Image Ret* consists of two separate datasets DIARETDB01 and DIARETDB1. DIARETDB1 consists of 89 fundus images with 5 healthy samples while the remaining samples have light symptoms of diabetic retinopathy such as hemorrhages, microaneurysms, hard exudates, and soft exudates. We used this dataset for the detection of hard exudate. On the other hand, STARE database was used for blood vessel segmentation.

Apart from general performance accuracy of the proposed approach, other performance metrics were considered. These metrics are similarity measures dependent on pixel-to-pixel template matching between the ground-truth template and its equivalent obtained using the proposed method. *True positive* (TP) and *true negative* (TN) are defined for correct classification. TP identifies all the candidate pixels that are correctly classified as candidates whereas the TN gives the number of noncandidate pixels that are correctly identified as noncandidate pixels. For misclassification, *false positive* (FP) and *false negative* (FN) are defined. FP is where a noncandidate pixel is misclassified as a candidate pixel whereas FN is where a candidate pixel is misclassified as noncandidate. The similarity measures considered are defined in Eqs. (14)–(19).

For example, true positive (TP) is computed as the number of white pixel intersection between ground-truth binary image and binary image obtained with our method whereas true negative (TN) is the number of black pixels in the intersection between ground-truth binary image and binary image obtained with our method. FP and FN are the number of white and black pixels in the complimentary set between the two templates, respectively.

(14) $$\begin{array}{c} \text{Sensitivity}=\frac{\left(\text{TP}\right)}{\left(\text{TP}+\text{FN}\right)\;}, \end{array}$$

(15) $$\begin{array}{c} \text{Specificity}=\frac{\left(\text{TN}\right)}{\left(\text{TN}+\text{FP}\right)\;}, \end{array}$$

(16) $$\begin{array}{c} \text{Accuracy}=\frac{\left(\text{TP}+\text{TN}\right)}{\left(\text{TP}+\text{FP}+\text{TN}+\text{FN}\right)\;}, \end{array}$$

(17) $$\begin{array}{c} \text{Positive prediction value},\text{PPV}=\frac{\left(\text{TP}\right)}{\left(\text{TP}+\text{FP}\right)\;}, \end{array}$$

(18) $$\begin{array}{c} \text{Negative prediction value},\text{NPV}=\frac{\left(\text{TN}\right)}{\left(\text{TN}+\text{FN}\right)\;}, \end{array}$$

(19) $$\begin{array}{c} \text{Structural similarity index},\text{SSIM}\left(x,y\right)=\frac{\left(2\mu_{x}\mu_{y}+c_{1}\right)\left(2\sigma_{xy}+c_{2}\right)}{\left({\mu^{2}}_{x}+{\mu^{2}}_{y}+c_{1}\right){\left({\sigma^{2}}_{x}+{\sigma^{2}}_{y}+c_{2}\right)}^{\prime }} \end{array}$$

where *μ*, *σ*, and *c*_1_ are the mean, standard deviation, and dynamic range constant of the template images *x* and *y*, respectively.

### 5.1. LSTM Implementation Information

**Figure 10:**
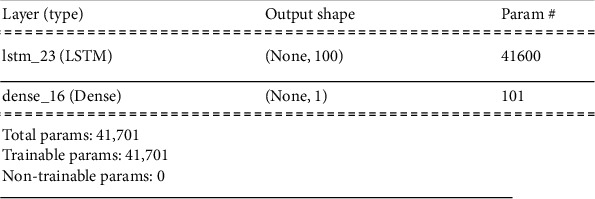
LSTM network model.

To realize the training of the LSTM network, python 3.8 programming language was used with TensorFlow and Keras as the libraries. Each cell of the LSTM has a look back memory of 3, meaning that to compute the present output of the cells, it uses three previous results from the preceded segments. The input layers are made of 100 cells, and the output layer (dense layers) is made of I neuron as indicated in the model summary in Figure 10. The network trained total of 41,701 parameters in 100 iterations with training and testing loss (RMSE) 20.79 and 30.13, respectively.

### 5.2. Hard-Exudate Detection

The output of the LSTM network determines the existence or nonexistence of hard exudates in a particular image segment based on the predicted *ρ* value. For *ρ* less or equals 0.5, it is assumed that hard exudate is absent in the segment. Values of *ρ* greater than 0.5 indicate presence of hard exudates but at different stages (*none*, *mild*, *strong*, and *very strong*).

**Figure 11:**
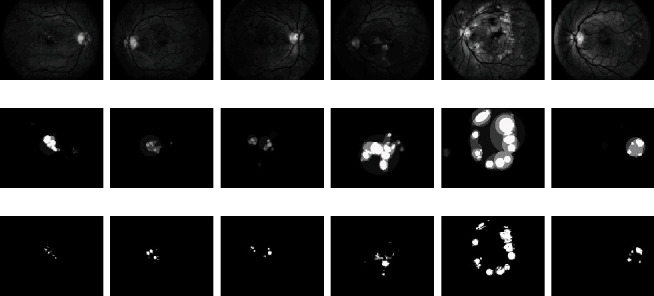
(a) Contains the preprocessed green channel of fundus image using CLAHE, (b) corresponding ground-truth images marked by experts with four categories of confidence level, and (c) results obtained using proposed technique.

However, to further classify pixels within a segment for template matching with the ground-truth image, further processing is required. Previously computed quantized version of the segment (*I*_8_, *I*_16_, *I*_64_, *I*_128_) is transformed using a nonlinear gamma transformation (Eq. (20)) to enhance their contrast. The constant gamma in the transformation is taken from the output score *ρ* of the segment from the LSTM network. These gamma-transformed segments are converted to binary image using Otsu global thresholding. Only segments with *ρ* > 0.5 are considered, and all pixels in segments with *ρ* ≤ 0.5 are classified as noncandidate. Pixels in the segments with *ρ* > 0.5 are classified by computing the intersection of the four different (quantized, gamma-transformed, and binarized) versions of the segment using Eq. (21).  Figure 11 and Table 1 present the extract from the experimental results on the DIARETDB1 database using the proposed technique.

(20) $$\begin{array}{c} I_{Q\left(i,j\right)}=\left\{\begin{array}{c} 1\text{ if}{cI}_{Q\left(i,j\right)}^{\left(1-\rho \right)}>\text{threshold},\\ 0\text{ otherwise}, \end{array}\right. \end{array}$$

where *c* is a constant of the gamma transform

(21) $$\begin{array}{c} I_{\text{candidate}}=\left|I_{8}\cap I_{16}\cap I_{32}\cap I_{64}\right|. \end{array}$$

### 5.3. Blood Vessel Segmentation

**Figure 12:**
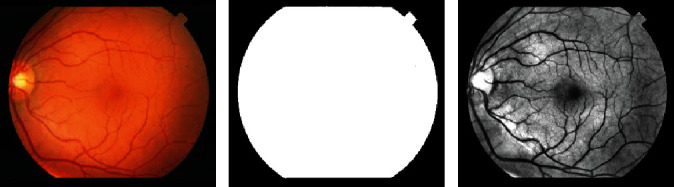
(a) Original RGB fundus image from STARE, (b) estimated background mask, and (c) quantized image (16 bins) with background removed.

**Figure 13:**
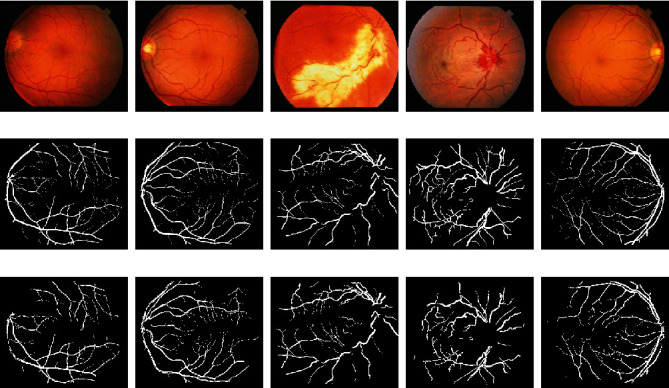
Segmentation of the vascular system. (a) Original images, (b) ground-truth manually annotated vascular system, and (c) results using the proposed approach.

As a sign of diabetic retinopathy, blood vessels may swell up and leak fluid leading to formation of abnormalities such as hard and soft exudates. In other situation, development of abnormal new blood vessels, blood vessel occlusion, and leakage of blood (hemorrhages) into a healthy portion of the retina are experienced. Detection of the signs of diabetic retinopathy involves the proper segmentation to give clue on any abnormal development on and around the vessels. The segmentation deploys the same approach as hard-exudate detection (using Eqs. (20) and (21)) except that a background estimation is used to remove background from the preprocessed image before quantization. To estimate the background, the three channels of the RGB image are sum up together and the resulting image is converted to binary using threshold of 100 values as shown in Figure 12.  Figure 13 presents the results of the segmentation of the blood vessel using the proposed method where in Table 2, a sample of performance measure results of automatic blood vessel segmentation in the STARE database is tabulated.

## 6. Comparison and Discussion

Detection of a particular symptom of diabetic retinopathy is quite a challenging task. This is due to the fact that some of these symptoms have similar textural composition and intensity distributions which makes it hard to differentiate using simple textural or intensity distribution analysis. For instance, in retina vasculature structure when viewed through the green channel of the fundus image, it exhibits intensity distribution similar to the optical disk which sits right on top of the nerve center where these vascular structures originate. Therefore, separating such instance needs more than just filtering but much more robust approach is needed to distinctively separate and scale up the tiniest of difference existing between these features. The use of different quantization level significantly enhances the possibility of differentiating overlapping features in the image. Different quantization level transforms the features into a different resolution whereby some features that are not visible in higher resolution (higher quantization level) suddenly become visible and therefore can easily be analyzed.

In detection of symptoms like blood hemorrhage and microaneurysm, they are usually tiny spots which often are too challenging because any noise in the processed image could take the form of these symptoms. The use of sequential training with LSTM becomes very handy and efficient since its output can be used to decide if a segment of the image has the symptom or not. These approaches ensure that a smoothen output is obtained, and only segments with higher probability of symptoms are further postprocessed. In Table 3, we present comparison results between the proposed approach and other state-of-the-art approaches.

## 7. Conclusions

A new approach for detecting symptoms of diabetic retinopathy has been proposed. An algorithm which thoroughly and comprehensively analyzes the retina vascular structure and hard exudate has been developed. The approach encodes in it, a powerful feature representation of the analyzed symptoms which facilitated improved performance compared to its counterparts in the literature. The use of different quantization levels transforms the spatial image domain to different resolution domains and significantly helps to improve on the interclass difference between features of similar textural and intensity distribution. Moreover, the LSTM model has been very effective and helps to mitigate the presence of noise or false positive occurrences in the final postprocessed output image. It also reduces time needed to postprocess the image as only segments with higher probability of symptoms cooccurrence are considered.

In summary, the results obtain are impressive and validate the relevance and the efficiency of the proposed approach in this context. Despite the success, the proposed method does not cover the detection of other symptoms of diabetic retinopathy like hemorrhage and microaneurysm. Other symptom detection might present different challenges.

## Figures and Tables

**Table 1:** Extract of performance measure results of hard exudates in DIARETDB1.

Image	Specificity (%)	Sensitivity (%)	SSI (%)	PPV (%)	NPV (%)	Accuracy (%)
Image01	98.50	76.78	99.59	57.66	96.25	98.19
Image02	94.89	85.01	98.60	42.69	96.94	92.43
Image03	98.56	88.76	99.44	66.59	98.36	98.54
Image04	86.67	82.16	99.46	54.71	96.26	86.63
Image05	93.99	98.14	98.23	70.19	96.99	94.02
Image06	95.95	95.61	99.57	61.85	99.26	95.95

**Table 2:** Extract of performance measure results of automatic blood vessel segmentation in STARE.

Image	Specificity (%)	Sensitivity (%)	SSI (%)	PPV (%)	NPV (%)	Accuracy (%)
Image01	94.34	70.54	99.38	55.31	96.99	92.19
Image02	82.03	91.84	98.55	27.67	99.26	82.71
Image03	92.88	86.88	99.47	51.54	98.78	92.40
Image04	92.79	78.70	99.37	46.83	98.18	91.74
Image05	94.13	85.30	99.55	55.36	98.68	93.43

**Table 3:** Comparisons with other methods.

Authors	Method	Accuracy of classification	Sensitivity	Specificity	Positive predictive value (PPV)
[22] Muhammad Faisal et al.	Support vector machınes (SVMs)	Not reported	96.9%	100%	100%
[23] R. Radha et al.	Morphologıcal process and clusterıng technıque	98%	Not reported	Not reported	Not reported
[24] Sumandeep Kaur et al.	K-means colour compression and fuzzy logic	96%	94.7%	Not reported	Not reported
[25] Akara Sopharak et al.	Using fuzzy C-means clustering	87.28%	99.24%	42.77%	24.26
[2] Acharya et al.	Blood vessel, exudates, microaneurysms, hemorrhages	86%	82%	86%	Not reported
[26] Vujosevic et al.	Single lesions	Not reported	82%	92%	Not reported
[27] R.H.N.G. Ranamuka et al.	Fuzzy logic	Not reported	75.43%	99.99%,	Not reported
[28] Pavle et al.	Deep neural networks and anatomical landmark	Not reported	78%	Not reported	78%
[14] T. Walter et al.	Means of morphological reconstruction techniques	92.8%	Not reported	Not reported	92.4%
[29] E. Imani et al.	Signal separation algorithm	89.01%	99.93%	82.64%	Not reported
[30] Abdullah Saeed et al.	Digital analysis and mathematical morphology operations	86%	80%	84.69%	Not reported
Proposed method	Local extrema quantized Haralick features with Long Short-Term Memory (LSTM) network	95.45%	91.65%	95.45%	99.34%

## Data Availability

The two datasets used in the research can be publicly accessed through the below links: (1) DIARETDB1: http://www2.it.lut.fi/project/imageret/diaretdb1_v2_1/ and (2) STARE: https://cecas.clemson.edu/~ahoover/stare/.

## References

[1] Meenu G., Sheifali G. (2016). Retinal blood vessel segmentation algorithms: a comparative. *International Journal of Bio-Science and Bio-Technology*.

[2] Acharya U. R., Lim C. M., Ng E. Y. K., Chee C., Tamura T. (2009). Computer-based detection of diabetes retinopathy stages using digital fundus images. *Proceedings of the Institution of Mechanical Engineers, Part H: Journal of Engineering in Medicine*.

[3] Onal S., Dabil-Karacal H. (2016). Improved automated vessel segmentation for diagnosing eye diseases using fundus images. *Journal of Biomedical Graphics and Computing*.

[4] N E Institute "National Eye Institute," National Eye Institute, 17 july 2019. https://www.nei.nih.gov/learn-about-eye-health/resources-for-health-educators/eye-health-data-and-statistics/diabetic-retinopathy-data-and-statistics.

[5] Kaur I., Singh L. M. (2016). A method of disease detection and segmentation of retinal blood vessels using fuzzy C-means and neutrosophic approach. *Imperial Journal of Interdisciplinary Research*.

[6] Chorage S. S., Khot S. S. (2016). A review on vessel extraction of fundus image to detect diabetic retinopathy. *Global Journal of Computer Science and Technology*.

[7] Besenczi R., Tóth J., Hajdu A. (2015). A review on automatic analysis techniques for color fundus photographs. *Computational and Structural Biotechnology Journal*.

[8] Yin Y., Adel M., Bourennane S. (2013). Automatic segmentation and measurement of vasculature in retinal fundus images using probabilistic formulation. *Computational and mathematical methods in medicine*.

[9] Ravichandran C. G., Raja J. B. (2014). A fast enhancement/thresholding based blood vessel segmentation for retinal image using contrast limited adaptive histogram equalization. *Journal of Medical Imaging and Health Informatics*.

[10] Wang S., Yin Y., Cao G., Wei B., Zheng Y., Yang G. (2015). Hierarchical retinal blood vessel segmentation based on feature and ensemble learning. *Neurocomputing*.

[11] Sohini R., Dara K., Keshab P. (2014). Blood vessel segmentation of fundus images by major vessel extraction and sub-image classification. *IEEE Journal of Biomedical and Health Informatics*.

[12] Zhao Y., Liu Y., Wu X., Harding S. P., Zheng Y. (2015). Retinal vessel segmentation: an efficient graph cut approach with retinex and local phase. *PLOS ONE*.

[13] Zhao Y., Rada L., Chen K., Harding S. P., Zheng Y. (2015). Automated vessel segmentation using infinite perimeter active contour model with hybrid region information with application to retinal images. *IEEE Transactions on Medical Imaging*.

[14] Walter T., Massin P., Erginay A., Ordonez R., Jeulin C., Klein J.-C. (2007). Automatic detection of microaneurysms in color fundus images. *Medical Image Analysis*.

[15] Spencer T., Olson J. A., McHardy K. C., Sharp P. F., Forrester J. V. (1996). An image-processing strategy for the segmentation and quantification of microaneurysms in fluorescein angiograms of the ocular fundus. *Computers and Biomedical Research*.

[16] Frame A. J., Undrill P. E., Cree M. J. (1998). A comparison of computer based classification methods applied to the detection of microaneurysms in ophthalmic fluorescein angiograms. *Computers in Biology and Medicine*.

[17] Haralick R. M., Shanmugam K., Its’Hak Dinstein (1973). Textural features for image classification. *IEEE Transactions on Systems, Man, and Cybernetics*.

[18] Verma M., Raman B., Murala S. (2015). Local extrema co-occurrence pattern for color and texture image retrieval. *Neurocomputing*.

[19] Hochreiter S., Schmidhuber J. (1997). Long Short-Term Memory. *Neural Computation*.

[20] Hoover A., Kouznetsova V., Goldbaum M. (2000). Locating blood vessels in retinal images by piece-wise threshold probing of a matched filter response. *IEEE Transactions on Medical Imaging*.

[21] Kauppi T., Kalesnykiene V., Kamarainen J. DIARETDB1 diabetic retinopathy database and evaluation protocol.

[22] Faisal M., Wahono D., Purnama K. W., Hariadi M., Purnomo M. H. (2014). Classıfıcatıon of dıabetıc retınopathy patıentsusıng support vector machınes (SVM) based on dıgıtal retınal ımage. *Journal of Theoretical and Applied Information Technology*.

[23] Radha R., Lakshman B. (2013). Retınal image analysis using morphological process and clustering technique. *Signal & Image Processing: An International Journal (SIPIJ)*.

[24] Kaur S., Singh D. Early detection and classification of diabetic retinopathy using K-means colour compression and fuzzy logic.

[25] Sopharak A., Uyyanonvara B., Barman S. (2009). Automatic exudate detection from non-dilated diabetic retinopathy retinal images using fuzzy C-means clustering. *Sensors*.

[26] Vujosevic S., Benetti E., Massignan F. (2009). Screening for diabetic retinopathy: 1 and 3 nonmydriatic 45-degree digital fundus photographs vs 7 standard early treatment diabetic retinopathy study fields. *American Journal of Ophthalmology*.

[27] Ranamuka N. G., Meegama R. G. N. (2012). Detection of hard exudates from diabetic retinopathy images using fuzzy logic. *PNCTM*.

[28] Prentašic P., Aric S. L. (2016). Detection of exudates in fundus photographs using deep neural networks and anatomical landmark detection fusion. *Computer Methods and Programs in Biomedicine*.

[29] Imani E., Pourreza H. (2016). A novel method for retinal exudate segmentation using signal separation algorithm. *Comput Methods Programs Biomed*.

[30] Saeed A., Alharthi A. M., Emamian V. (2016). An automated mechanism for early screening and diagnosis of diabetic retinopathy in human retinal images. *British Journal of Applied Science & Technology*.

